# Risk of endometrial cancer after RRSO in *BRCA 1/2* carriers: a multicentre cohort study

**DOI:** 10.1007/s12094-023-03312-4

**Published:** 2023-09-08

**Authors:** Helena Pla-Juher, Marta Pardo, Àngel J. Izquierdo, Esther Darder, Anna Carbó, Elisabet Munté, Sara Torres-Esquius, Judith Balmaña, Concepción Lázaro, Joan M. Brunet, Maria-Pilar Barretina-Ginesta

**Affiliations:** 1 Medical Oncology, Catalan Institute of Oncology, Hospital Universitari Dr. Josep Trueta, Avinguda de França s/n, 17707 Girona, Spain; 2 Precision Oncology Group (OncoGIR-Pro), Institut d’Investigació Biomèdica de Girona (IDIBGI), Girona, Spain; 3 Hereditary Cancer Program, Catalan Institute of Oncology, IDIBGI, Girona, Spain; 4 Department of Epidemiology and Cancer Register, Girona, Spain; 5 Hereditary Cancer Program, Catalan Institute of Oncology, IDIBELL, L’Hospitalet, Barcelona, Spain; 6 Department of Medical Oncology, Vall d’Hebrón Hospital, Barcelona, Spain; 7 Centro de Investigación Biomédica en Red de Cáncer (CIBERONC), Instituto Salud Carlos III, Madrid, Spain

**Keywords:** Endometrial cancer, Breast cancer, Risk-reducing salpingo-oophorectomy, *BRCA*, Mutation, Hysterectomy, Tamoxifen, Carcinosarcoma, Serous carcinoma, Endometrioid carcinoma

## Abstract

**Objective:**

To know the risk of endometrial cancer (EC) in a population of women with *BRCA 1/2* pathogenic or likely pathogenic variants after risk-reducing salpingo-oophorectomy (RRSO).

**Methods:**

The study cohort included data from 857 women with *BRCA* mutations who underwent RRSO visited four hospitals in Catalonia, Spain, from January 1, 1999 to April 30, 2019. Standardized incidence ratio (SIR) of EC was calculated in these patients using data from a regional population-based cancer registry.

**Results:**

After RRSO, eight cases of EC were identified. Four in *BRCA 1* carriers and four in *BRCA2* carriers. The expected number of cases of EC was 3.67 cases, with a SIR of 2.18 and a 95% CI (0.93–3.95).

**Conclusions:**

In our cohort, the risk of EC in *BRCA1/2* carriers after RRSO is not greater than expected. Hysterectomy is not routinely recommended for these patients.

## Introduction

Women who are germline *BRCA* 1/2 mutation carriers are at an increased risk of developing tumours, specially breast and ovarian/fallopian tube cancers, as compared with the general population. It is well-studied that these patients have a lifetime risk of ovarian cancer of 39–46% if they carry the *BRCA1* mutation and 12–20% if they carry the *BRCA2* mutation [1].

The estimated prevalence of *BRCA1* and *BRCA2* mutations is dependent on the population and can vary between 1 in 300 and 1 in 800, respectively, being higher in populations such as Ashkenazi Jewish [1, 2].

Nowadays, genetic testing for *BRCA* is recommended in women diagnosed with ovarian cancer regardless of their family history as they might benefit from PARP inhibitor therapies. This fact can increase the detection of *BRCA* carriers which can, therefore, lead to changes in treatment decisions regarding prophylactic surgeries.

Current guidelines recommend undergoing risk-reducing salpingo-oophorectomy (RRSO) by the age of 35–40 for *BRCA1* and by the age of 40–45 for *BRCA2* and once child-bearing is complete to reduce the risk of ovarian or fallopian tube cancer by 80–90% and also to reduce mortality in patients harbouring these mutations [2–8]. However, hysterectomy at the same time as RRSO is controversial.

Such information remains of clinical importance since endometrial cancer is the most common gynaecological malignancy, yet it is still unknown if there is any increased risk in women with *BRCA* mutations. Moreover, it must be considered that endometrial cancer in early stages has a good prognosis while survival in locally advanced or metastatic setting is nowadays very limited, with a 5 years survival rate of approximately 47% [9]. In addition, its prognosis may change with the prophylactic surgery if added benefit of hysterectomy had been confirmed. Furthermore, if the association of an increased EC risk in *BRCA* carriers had been confirmed, it might influence clinical implications such as genetic counselling and treatment strategies.

Regarding this topic, different articles have been published with discordant results. Few authors have recommended concomitant hysterectomy because there might be a high risk of EC among patients carrying a *BRCA* mutation. Moreover, a study [10] has suggested an association between *BRCA1* and serous endometrial cancer subtype. Nevertheless, outcomes of other studies have not confirmed this [11–14].

In addition, there have also been different findings related to the EC cancer risk possibly confounded by the use of tamoxifen, an adjuvant therapy for hormone receptor-positive breast cancer [15–18].

The goal of our analysis was to estimate the incidence of endometrial cancer among a cohort of women with *BRCA* mutations who underwent risk-reducing salpingo-oophorectomy compared to general population to assess the benefit of undergoing hysterectomy in the RRSO setting.

## Material and methods

### Study cohort

Data from 2,237 women with *BRCA* mutation, 857 of which underwent RRSO without a prior hysterectomy, visited four hospitals in Catalonia (Spain) from January 1, 1999 to April 30, 2019 were analyzed. Data were obtained from the Hereditary Cancer Registry that contains data ambispectively collected. Participants were followed-up until January 1, 2023 for a median of 7.13 (IQR 6.31) years after *BRCA* testing. Censoring occurred at either endometrial cancer diagnosis, last follow-up or death.

The cohort inclusion criteria were (a) positive *BRCA1* or *BRCA2* mutation test results, (b) aged 18 or older (c) no previous history of endometrial cancer and (d) risk-reducing salpingo-oophorectomy.

Information related to age, EC histology and stage, previous history of breast cancer (BC), treatment with tamoxifen and its duration, dates of EC diagnoses and type of surgery were collected from each patient through a review based on medical histories, surgical and pathology reports.

All patients have signed a written consent which has been approved by the local Ethical Committee and conducted in accordance with the guidelines for Good Clinical Practice and the Declaration of Helsinki.

### Statistical analysis

To compare the risks with the general population, the standardized incidence ratios were calculated by dividing the number of observed endometrial cancer cases by the number of expected ones. The number of expected cases was calculated taking into account the incidence rate standardized by age of EC of the general population in Catalonia, from 2008 to 2018 that was 21.5 new cases per 100,000 women/year. Data were obtained from the Population-based Cancer Registry database.

## Results

A total of 2,237 *BRCA1/2*+ patients were selected. Among them 1,122 (49.36%) were *BRCA*1 mutated and 1,115 (49.84%) *BRCA2*. Eight hundred fifty-seven 857 (37%) underwent RRSO without hysterectomy (423 (49.35%) were *BRCA1* and 434 (50.64%) *BRCA2*) and were included in the analyses.

After RRSO, eight cases (0.93%) of EC were identified. Four of them in *BRCA1* and four in *BRCA2*. The characteristics of these EC were as follows (Table 1): four were endometrioid, three were serous and one was a carcinosarcoma. Two of the three serous subtypes were in patients *BRCA1* mutated. Six were stage IA according to the International Federation of Gynaecology and Obstetrics (FIGO) classification, one was stage IIIA and one IIIC1. The median age was 52 years old (range 45–70). Between the date of RRSO and EC diagnosis, 1–3 years had passed in three cases and 3–6 years in four patients whereas 13 years was the time passed in one case.

**Table 1:** Characteristics of patients who developed EC

Case	Mutation status	Histology of EC	FIGO stage	Age at DX of BC and start of TMX (years)	Duration of TMX treatment (years)	Menopausal status	Surgical state	Age at DX of EC (years)
1	BRCA 1	Serous	IA	–	–	–	RRSO	65
2	BRCA 1	Serous	IA	43	2	Pre-	RRSO	46
3	BRCA 1	Endometrioid	IA	–	–	–	RRSO	45
4	BRCA 1	Carcinosarcoma	IA	–	–	Post-	RRSO	53
5	BRCA 2	Endometrioid	IA	–	–	Post-	RRSO	69
6	BRCA 2	Endometrioid	IA	32	5	Pre-	RRSO	51
7	BRCA 2	Endometrioid	IIIA	–	–	–	RRSO	56
8	BRCA 2	Serous	IIIC1	42	2	Pre-	RRSO	51

*EC* endometrial cancer, *BC* breast cancer, *TMX* tamoxifen, *RRSO* risk-reducing salpingo-oophorectomy

Six out of eight patients had a history of breast cancer, and of these, three had taken tamoxifen previously (one case for 5 years and the others for 2 years). Of those who had taken TMX, the interval between breast cancer diagnosis and endometrial cancer was 3.9–19 years. One of these had serous histology and *BRCA1* mutation, and the other two had endometrioid and serous histology with a *BRCA2* mutation.

In our analyses, expected EC in the cohort studied would be 3.67 cases, being our overall SIR 2.18 with a 95% CI (0.93–3.95). Expected cases separately for *BRCA1* and *BRCA2* carriers were 1.82 and 1.87, respectively, being the standardized incidence rates 2.2 (95% CI 0.57–4.88) and 2.14 (95% CI 0.49–4.15) respectively, resulting in an incidence rate ratio for *BRCA2*–*BRCA1* of 0.97 (95% CI 0.23–4.01).

## Discussion

### BRCA mutation and endometrial cancer

Several articles have been published evaluating the impact of RRSO on *BRCA*-associated cancer risk [2, 3, 5, 19] due to the importance of reducing ovarian, fallopian tube, peritoneal and breast cancers established in these patients as well as increasing survival.

At the time of risk-reducing surgery, both ovaries and fallopian tubes are removed, but there is controversy as to whether a hysterectomy should be performed concomitantly owing to a possible strong association between endometrial cancer and *BRCA* mutations which remains to be demonstrated.

The contribution of *BRCA* mutations to endometrial cancer has been reported in some publications, such as that of Kauff et al*.* [3] who reported the risk of EC arose from the small amount of intramural fallopian-tube tissue that is remains after the salpingo-oophorectomy. Furthermore, a published article by Shu *et*. al [10] suggested an increased risk of serous endometrial cancer in *BRCA1* mutation carriers (O:E ratio 22.2; 95% CI, 6.1–56.9; *p* < 0.001). Similar results were shown in recent studies where *BRCA 1* and *2* carriers have an increased risk of serous endometrial carcinoma and prophylactic hysterectomy could be discussed for this population [6, 22, 23]. A study published by De Jonge et al*.* demonstrated an increased risk of EC in patients with germline *BRCA*-associated hereditary breast and ovarian cancer syndrome. Those patients with loss-of-heterozygosity were more likely to be diagnosed with non-endometrioid and histological grade 3 endometrial cancer [20]. In 2021, a paper of a Dutch nationwide cohort study described a two-to-threefold increased risk for EC in *BRCA 1/2* mutation carriers, especially for rare histological subgroups such as serous-like and p-53 abnormal EC [21].

Nevertheless, a recent systematic review has identified all relevant studies regarding *BRCA* mutation and EC. Only 5 out of 14 studies have found a correlation between *BRCA* mutation and an increased risk of EC [23]. Hence, this does not provide evidence in favour of performing a routine hysterectomy at the time of RRSO.

In line with these findings, our results indicate there is no evidence that carrying the *BRCA1* mutation gives a higher likelihood for developing EC than carrying the *BRCA2* mutation (0.97 95% CI 0.23–4.01).

### Tamoxifen and endometrial cancer

Tamoxifen is a selective oestrogen receptor modulator. It has an anti-oestrogenic effect on breast tissue while it acts as an oestrogenic factor in endometrium. It has been widely used as adjuvant treatment for breast cancer.

It is important to take into account the role of tamoxifen as it is a known risk for endometrial cancer [15, 16] specifically in postmenopausal patients [18]. Multiple studies have shown an increased risk of endometrial cancer in association with prolonged tamoxifen use. Some authors published an increased risk of aggressive histology of endometrial cancer with poor prognosis in those treated with tamoxifen [18, 25] while others have not found this relationship [14, 24].

Segev et al. [16] found that *BRCA1* mutation carriers with a history of tamoxifen use had a significant increase in the incidence of endometrial cancer (O:E ratio 4.43; 95% CI, 1.94–8.76). Additionally, Wen et al*.* [25] reported that those women with decreased *BRCA1* protein expression treated with tamoxifen might be more susceptible to serous endometrial cancer. Whereas some studies have not confirmed this information [12], there are authors who support that hysterectomy should be suggested only to those likely to use tamoxifen [16] and taking into account that this surgical procedure has no negligible risks [26].

In our cohort, three out of eight EC patients had been treated with tamoxifen due to a previous diagnosis of breast cancer, indicating that it could be one of the risk factors that contributed to the development of EC. One of them was *BRCA1* and two *BRCA2* mutation carriers. However, none of them were postmenopausal, as expected based on previous studies.

### Hysterectomy and endometrial cancer

Despite being less with minimally invasive procedure, risks and benefits of prophylactic hysterectomy should be weighed and discussed with patients to address issues of quality of life and morbi-mortality of this intervention [4]. It is known that performing concomitant surgeries increases morbidity and complications such as pelvic organ prolapse and urinary incontinence, and it may result in sexual dysfunction. However, advantages of concomitant hysterectomy at the time of RRSO should be balanced regarding management of menopausal symptoms with hormone replacement therapy (HRT). Combined HRT with oestrogen and progestin are required when the uterus is left in place because unopposed oestrogen increases EC risk, but combined HRT may further increase the risk of BC which is important to *BRCA* carriers [23].

Based on the results of Shu et al., Havrilesky and colleagues [26] estimated the mortality reduction benefit and cost-effectiveness of performing a hysterectomy at the time of RRSO to prevent serous uterine cancer in *BRCA1* carriers in the United States. They suggested that hysterectomy is highly cost-effective with an increased life expectancy of 5 months.

Furthermore, three population-based studies reported mortality due to hysterectomy of 0.03–0.06% which is much lower compared to the estimate risk for serous uterine cancer in patients aged up to 70 years old of 2.6% in *BRCA1* carriers [10].

The strengths of our study are the high number of patients from a multicentre ambispective cohort that include clinical data collected from medical and pathology reports. However, we recognize a few limitations such as data on risk factors (epidemiological, hormone replacement therapy or lifestyle) associated with EC that does not allow for drawing conclusions.

## Conclusions

This study revealed that there was no significantly higher risk of endometrial cancer in *BRCA*-mutation carriers, and despite some literature suggesting an association between *BRCA1* and serous endometrial cancer, this study does not reinforce this association. Further large studies are required to better estimate the risk of EC before hysterectomy at the time of RRSO and whether it is justifiable for women harbouring *BRCA* mutations. A multidisciplinary approach should be offered to these patients to assess the best risk-reducing options.
